# IL-13 Contributes to Drug Resistance of NK/T-Cell Lymphoma Cells by Regulating ABCC4

**DOI:** 10.1155/2018/2606834

**Published:** 2018-12-13

**Authors:** Mingli Ni, Beibei Qin, Ling Xie, Xudong Zhang, Jiezhi Yang, Hongqiong Lv, Mingyue Yang, Mingzhi Zhang

**Affiliations:** ^1^Department of Oncology, the First Affiliated Hospital of Zhengzhou University, Zhengzhou 450052, China; ^2^Department of Oncology, Luoyang Central Hospital Affiliated to Zhengzhou University, Luoyang 471009, China

## Abstract

**Background:**

Extranodal natural killer/T (NK/T) cell lymphoma, nasal type (ENKTL), represents a rare subtype of T-cell lymphomas with aggressive clinical behavior and is relatively resistant to chemotherapy. However, there is relatively poor understanding of molecular pathogenesis of multidrug resistance in ENKTL. Here, we aimed to explore the biological roles and potential mechanism of IL-13 and ABCC4 in multidrug resistance of NK/T-cell lymphoma.

**Methods:**

ELISA analysis was used to determine the level of serum IL-13 and immunohistochemical analysis was applied to detect the ABCC4 expression level in patients with human NK/T-cell lymphoma. Western blot assay was employed to measure the expression of ABCC4 in cells. Lenti-sh-ABCC4 viruses were constructed to knock down ABCC4 in YTS cells. CCK-8 assay and flow cytometric analysis were performed to detect the effects of IL-13 and ABCC4 on cell proliferation and apoptosis. CCK-8 assay was conducted to detect the effect of IL-13 and ABCC4 on cell sensitivity to adriamycin (ADM) in YTS cells.

**Results:**

Levels of serum IL-13 and ABCC4 expression were observed to be upregulated in patients with human NK/T-cell lymphoma. Moreover, ABCC4 protein expression was also increased in NK/T-cell lymphoma YTS cells compared to the normal NK cells. Interestingly, IL-13 promoted ABCC4 expression in YTS cells. IL-13 promoted proliferation and suppressed apoptosis of YTS cells and reversed the effects of ABCC4 knockdown on promotive proliferation and inhibitory apoptosis. In addition, IL-13 enhanced YTS cell chemotherapy resistance to ADM by promoting ABCC4 expression.

**Conclusion:**

Our findings concluded that IL-13 inhibited chemotherapy sensitivity of NK/T-cell lymphoma cells by regulating ABCC4, disrupting which may effectively improve the therapy protocols against resistant NK/T-cell lymphoma.

## 1. Introduction

Extranodal natural killer (NK)/T cell lymphoma, nasal type (ENKTL), is an aggressive and rare Epstein-Barr virus- (EBV-) associated non-Hodgkin lymphoma that typically occurs in the naso/oropharynx [1]. ENKTL possesses the characteristic of high rates of systemic relapse and poor survival [2]. Currently, the clinical outcome for patients receiving chemotherapy or combined with radical radiotherapy is still unsatisfactory. Therefore, the recurrent problem of therapeutic resistance subdues needs to be urgently solved in this field.

Interleukin-13 (IL-13), predominantly a Th2-derived cytokine, plays an important role in fibrosis, inflammation, tissue hyperresponsiveness, and tumor development [3–5]. A report has illustrated that high systemic levels of IL-13 are related to the increases in the occurrence of different cancers [6]. A previous study has revealed that distinct cellular sources of IL-13, as well as precise targets of IL-13 that contribute to tumor progression, focus on both cells of hematopoietic lineage as well as epithelial and stromal cells [7]. In chemoresistant cells, the autocrine production of STAT3-target and IDO1-inducers cytokines IL-6, IL-4, IL-1*β*, IL-13, TNF-*α*, and CD40L which sustain the transcription of IDO1 in multidrug resistant cells is increased [8]. A report has shown that clinically half patients with EBV + T/NK-cell neoplasms have fever, which is possibly associated with inflammatory response syndrome induced by cytokines including IL-13 [9]. However, the effects and potential molecular mechanism of IL-13 in drug resistance of ENKTL remain unclear.

Multidrug resistance (MDR) is often associated with the increased expression of drug efflux transporters of the ATP-binding cassette (ABC) protein family, which can export a variety of chemotherapeutics out of the cells [10]. The ABC transporter superfamily contains 48 ABC transporters, which are further divided into seven subfamilies (ABCA-ABCG) based on sequence homology and protein organization [11]. Cumulative evidence has pointed out that the ABC transporters play pivotal roles in physiological transport and export of drugs and toxic substances, which are transported across membranes in an ATP-dependent manner [12–14]. ABCC4, located on chromosome 13q32.1, encodes for the 1325-amino acid-long human ABCC4 (MRP4) and is an important member of the ATP-binding cassette transporter family [10]. ABCC4 is widely expressed in various tissues, including the liver, kidney, ovary, and blood cells [10]. A study has revealed that downregulation of ABCC4 increases sensitivity to neoadjuvant chemoradiotherapy and longer disease-free survival in rectal cancer patients has been demonstrated [15, 16], which suggested that ABCC4 might be a possible predictive biomarker for the efficacy of chemotherapy in rectal carcinoma. However, the roles and potential mechanism of ABCC4 in multidrug resistance of ENKTL remain unclear.

The aim of our study was to explore the roles and potential mechanism of the cytokine IL-13 and resistance protein ABCC4 in ENKTL. We first revealed that IL-13 promoted NK/T-cell lymphoma cells chemotherapy resistance to adriamycin (ADM) by increasing ABCC4 expression. Our study provides a novel clue to explore promising therapy method for ENKTL.

## 2. Materials and Methods

### 2.1. Patients and Specimens

15 cases of human ENKTL tissues and 12 cases of rhinitis tissues specimens embedded in paraffin were obtained from patients who were admitted to the First Affiliated Hospital of Zhengzhou University during January 2015 and December 2016. Blood samples were harvested in serum separation tubes. After separation, the serum was stored at -80°C until analysis by enzyme linked immunosorbent assay (ELISA). All the study and protocols have been approved by the Ethics Committee of the First Affiliated Hospital of Zhengzhou University. The informed consent was obtained from all individual patients involved.

### 2.2. ELISA Analysis

Serum was stored at -80°C until analysis for IL-13 using ELISA kit purchased from Shanghai Bogu Biotechnology Co., Ltd. (Shanghai, China) according to the instructions.

### 2.3. Immunohistochemical Analysis

The paraffin-embedded tissue samples of human ENKTL tissues and rhinitis tissues were stained for ABCC4 with rabbit ABCC4 antibody (1:200, Cell Signaling Technology, Beverly, MA, USA) using standard immunohistochemistry method. The specific methods referred to the previous study [17]. Visual signals were classified into 4 grades of intensity as follows: (-) negative; (+) weakly positive, (++) moderately positive, and (+++) strongly positive.

### 2.4. Cell Culture

The normal NK cells were purchased from Sangon Biotech (Shanghai, China) and cell culture program was kept strictly confidential by Sangon Biotech company. Human natural killer (NK) lymphoma cell line YTS, established from primary lesions with nasal NK/T-cell lymphoma, was kindly provided by Dr. Daniel Billadeau from the Mayo Clinic (Rochester, MN, USA). YTS cells were cultured in RPMI-1640 (Gibco-Invitrogen) including 10% FBS (FBS, Takara Biotechnology Co., Ltd., Dalian, China), penicillin G (100 units /ml), and 1% penicillin-streptomycin (100 *μ*g/ml; Invitrogen Life Technologies, Beijing, China) at 37°C in 5% CO_2_.

HEK 293T cells (ATCC, Manassas, VA, USA) were cultured in Dulbecco's modified Eagle's medium (DMEM, Hyclone, Logan, UT) with 10% FBS and 1% penicillin-streptomycin at 37°C in 5% CO_2_.

### 2.5. Construction of the Lenti-sh-ABCC4 Vector and Lentivirus Packaging

To investigate effects of ABCC4 on the biological functions in human ENKTL YTS cells, we constructed 2 RNA interference lentiviral vectors aimed at* ABCC4* gene. Targeting ABCC4 mRNA coding sequence, we designed two specific short hairpin RNAs (shRNAs) and constructed the lentiviral vectors (sh-ABCC4-1 and sh-ABCC4-2). The lentiviral vector was pLVX-shRNA1 which contains a puromycin resistance gene in this study. The packaging plasmids were pCMV-VSVG and pCMV-Δ8.2 expression plasmids.

HEK293T cells were seeded at 50-60% confluency, incubated overnight and cotransfected with 9 *μ*g of lentiviral vector, 6 *μ*g of pCMV-VSVG, and 3 *μ*g of pCMV-Δ8.2 expression plasmids using Lipofectamine 2000 (Invitrogen, Carlsbad, CA, USA) according to the manufacturer's instructions. After 48 h posttransfection, viral supernatants were harvested by centrifugation at 3000 rpm for 5 min at 4°C, clarified through a 0.22-*μ*m-pore-size membrane (Millipore) to remove cellular debris, and used immediately or stored at -80°C prior to infection. The high-concentration lentiviral concentrate was used to infect the YTS cells.

### 2.6. Lentiviral Transfection of the YTS Cells

YTS cells were plated in 24-well plates (4 × 10^4^ cells/well). The virus particles (lentivirus-carring vector and recombinant lentivirus-carring sh-ABCC4-1 or sh-ABCC4-2) were added into the cells at a density of 70%-80%, respectively. Cells were drug-selected using 4 *μ*g/mL puromycin to obtain the stably transfected cells. The transfection ratio was identified by western blot analysis. The cells demonstrated high transfection efficiencies served as the target cells.

### 2.7. Western Blot Assay

Total proteins from treated cells were extracted using RIPA lysis buffer (Santa Cruz Biotechnology Inc., Santa Cruz, CA, USA). The protein concentrations in the cell lysates were conducted using a BCA protein assay reagent (Pierce, Rockfold, IL, USA). The equal amounts of total protein (50 ng) were subjected to sodium dodecyl sulfate polyacrylamide gel electrophoresis (SDS-PAGE) and then transferred onto polyvinylidene fluoride membranes (PVDF, Sigma-Aldrich, Corp., Cambridge, UK). The immune blots were blocked with 5% skim milk for 1 h, and the blots were incubated with the primary antibodies against ABCC4 and *β*-actin (Cell Signaling Technology). Subsequently, the secondary antibody conjugated to HRP (Cell Signaling Technology) was added, and the blots were incubated at 37°C for 1 h. After washing twice with Tris-buffered saline plus Tween20 (TBST), the signals were visualized with an enhanced chemiluminescence detection kit (Amersham Pharmacia Biotech, Piscataway, NJ). The images were analyzed using the software program Image Gauge (LAS-1000plus, Fujifilm, Tokyo, Japan).

### 2.8. CCK-8 Assay

YTS cells were seeded at a density of 2×10^4^ cells per well in 96-well plates. YTS cells were treated with IL-13 (50 ng/ml). Cells were cultured at 37°C, 5%CO_2_ for the indicated times (1, 2, 3, 4, and 5 days) and then treated with 10 *μ*l of CCK-8 solution (Dojindo Molecular Technologies, Gaithers burg, MD, USA) at the indicated times. Cells were cultured for another 3 h. The amount of formazan dye generated by cellular dehydrogenase activity was measured by absorbance at 450 nm with a microplate reader (Enspire Multimode Plate Reader, PerkinElmer, USA).

### 2.9. Flow Cytometric Analysis

YTS cells were seeded in 6-well plates at 5 × 10^4^ per well. After treatments, cells were harvested by centrifugation and cell apoptosis was detected using the Annexin V-FITC/PI kit (Dojindo, Tokyo, Japan) according to the manufacturer's instructions. Briefly, the harvested cells were washed twice in cold PBS. 1 × 10^6^ cells were resuspended in 500 *μ*l binding buffer. The suspension was stained with 10 *μ*l Annexin V-FITC in the dark for 15 min at room temperature. And then, 10 *μ*l of PI was added to the suspension and the cells were incubated for 10 min at room temperature in the dark. The final analysis was performed by flow cytometry (Becton Dickinson FACS Calibur).

### 2.10. Statistical Analysis

All data are presented as means ± SD. Comparisons between two groups were evaluated by the* t*-test, while comparisons among three or more groups were evaluated by one-way ANOVA. The* P* value < 0.05 was considered significant.

## 3. Results

### 3.1. High IL-13 and ABCC4 Expression Levels Were Observed in ENKTL Patients

**Figure 1:**
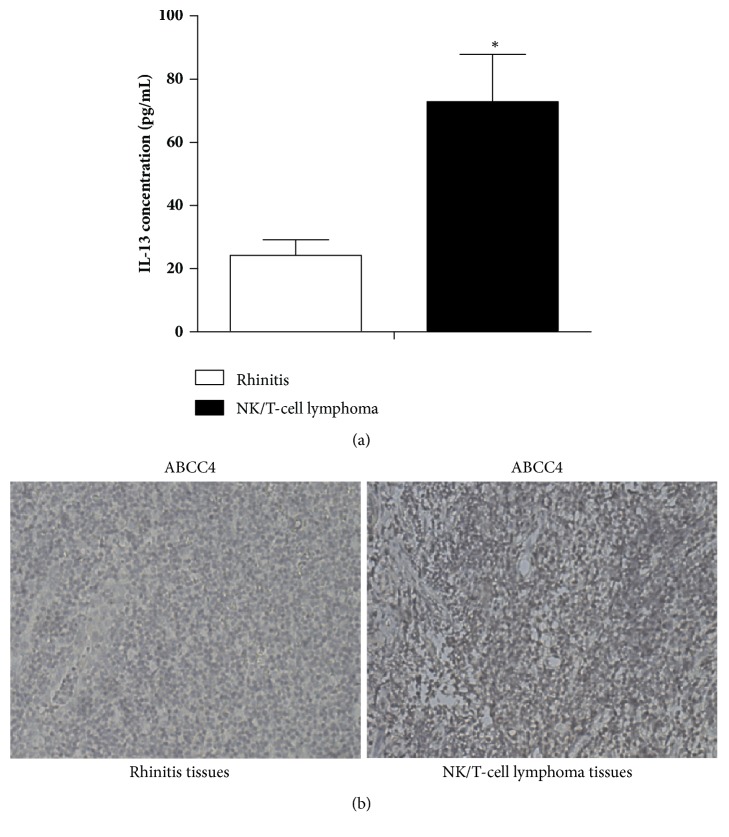
High serum IL-13 and ABCC4 expression levels were observed in NK/T-cell lymphoma patients. (a) ELISA assay was applied to measure the level of serum IL-13 in NK/T-cell lymphoma and rhinitis patients. (b) Immunohistochemical analysis was performed to detect the expression level of ABCC4 in NK/T-cell lymphoma tissues and rhinitis tissues (original magnification, ×200). *∗P* < 0.05.

**Figure 2:**
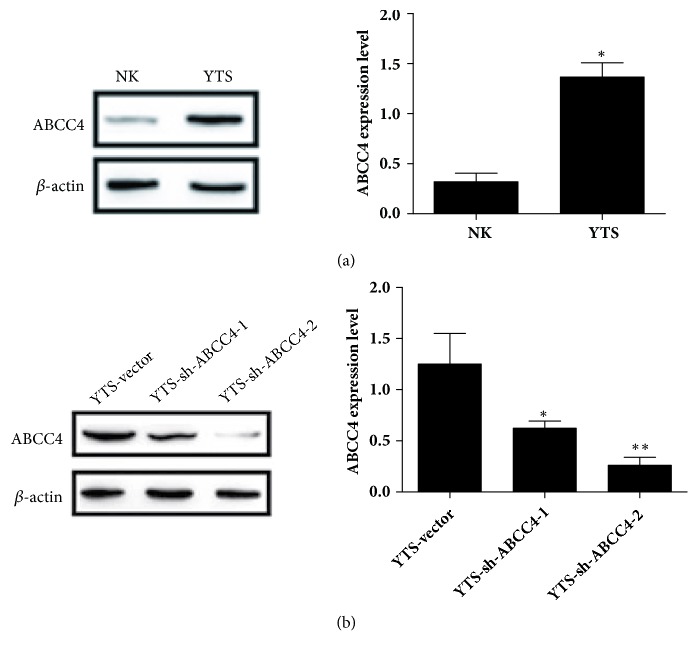
Expression of ABCC4 in YTS cells. (a) The expression of ABCC4 in NK and YTS cells was detected by western blot assay. (b) The expression level of ABCC4 in YTS cells transfected with or without sh-ABCC4-1 and sh-ABCC4-2. *∗P* < 0.05, *∗∗P* < 0.01.

ELISA and immunohistochemical and western blot analysis were performed to detect the IL-13 and ABCC4 expression levels, respectively.  Figure 1(a) showed that serum IL-13 level was significantly higher in patients with ENKTL than that in rhinitis group. ABCC4 expression level was influentially increased in ENKTL tissues compared with rhinitis tissues (Figure 1(b)). Moreover, results from western blot analysis revealed that there was also a marked rise in level of ABCC4 in ENKTL YTS cells than that in normal NK cells (Figure 2(a)). According these data, we speculated that IL-13 and ABCC4 expression levels were associated with the occurrence of multidrug resistance of ENKTL.

### 3.2. Knockdown of ABCC4 in Transfected YTS Cells

To further investigate the effects of ABCC4 on resistance of ENKTL YTS cells, we constructed the stable sh-ABCC4-YTS cells, in which ABCC4 expression was obviously reduced compared with control group. As Figure 2(b) has shown, ABCC4 expression was obviously reduced in YTS cells transfected with sh-ABCC4-1 and sh-ABCC4-2. The knockdown efficiency of sh-ABCC4-2 was higher than sh-ABCC4-1. Therefore, sh-ABCC4-2-YTS cells were used for the follow-up experiments.

### 3.3. IL-13 Promoted ABCC4 Expression in YTS Cells

**Figure 3:**
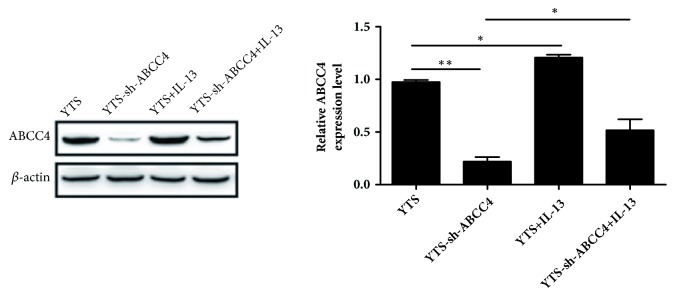
IL-13 promoted ABCC4 expression in YTS cells. The expression of ABCC4 was determined by western blot in YTS cells treated with or without IL-13, sh-ABCC4, and IL-13 + sh-ABCC4. *∗P* < 0.05, *∗∗P* < 0.01.

Next, to determine whether IL-13 could affect the expression of ABCC4, western blot assay was applied to measure the expression levels of ABCC4 in YTS cells. As shown in Figure 3, IL-13 treatment (50 ng/ml) significantly increased the expression of ABCC4 in YTS cells. In addition, IL-13 treatment could inverse the influence of sh-ABCC4 treatment on ABCC4 expression in sh-ABCC4-YTS cells. According to these results, we speculated that IL-13 might affect resistance of YTS cells via modulating ABCC4 expression.

### 3.4. IL-13 Promoted Proliferation and Suppressed Apoptosis of YTS Cells by Promoting ABCC4 Expression

**Figure 4:**
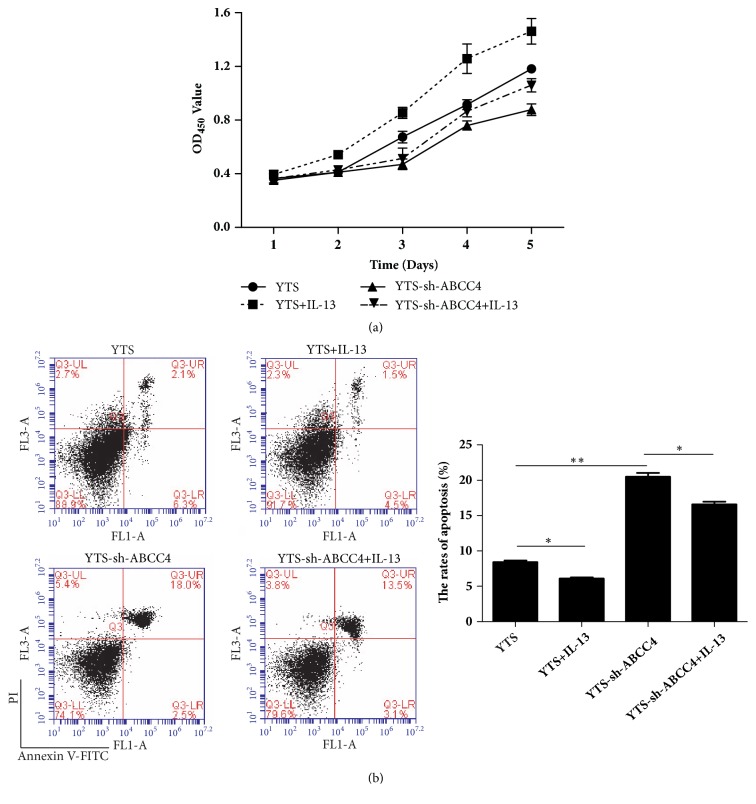
IL-13 promoted proliferation and suppressed apoptosis in YTS cells by promoting ABCC4 expression. (a) CCK-8 assay was conducted to determine cell proliferation of YTS cells treated with or without IL-13, sh-ABCC4, and IL-13 + sh-ABCC4 at day 1, 2, 3, 4, and 5. (b) Flow cytometric analysis was performed to detect the apoptosis rates of YTS cells treated with IL-13, sh-ABCC4, and IL-13 + sh-ABCC4 at 48 h. *∗P* < 0.05 and *∗∗P* < 0.01.

Further, to clarify the biological function of IL-13 and ABCC4 in ENKTL YTS cells, CCK-8 assay and flow cytometric analysis were carried out. The results from CCK-8 assay showed that IL-13 significantly promoted cell proliferation of YTS cells and knockdown of ABCC4 obviously suppressed cell proliferation, while IL-13 treatment could inverse the influence of knockdown of ABCC4 on proliferation of YTS cells. The results from flow cytometric analysis showed that IL-13 markedly reduced apoptosis rate of YTS cells, while ABCC4 downregulation could induce apoptosis in YTS cells (Figure 4(b)). Interestingly, IL-13 could overturn the effect of ABCC4 downregulation on apoptosis. These data suggested that IL-13 promoted proliferation and suppressed apoptosis of human ENKTL YTS cells by enhancing ABCC4 expression.

### 3.5. IL-13 Promoted YTS Cell Chemotherapy Resistance to ADM by Promoting ABCC4 Expression

**Figure 5:**
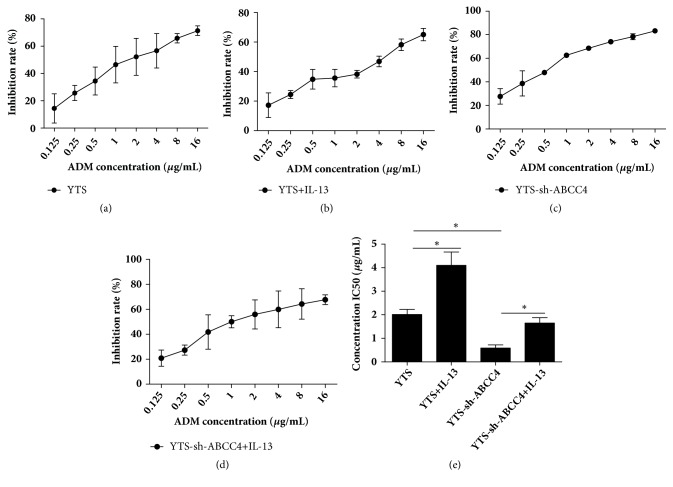
IL-13 promoted YTS cell chemotherapy resistance to ADM by enhancing ABCC4 expression. (a-d) The effect of IL-13 and ABCC4 on cytotoxicity of ADM to YTS cells treated with or without IL-13, sh-ABCC4, and IL-13 + sh-ABCC4 was demonstrated by CCK-8 at 72 h. (e) The IC50 value for ADM was calculated by graphPad Prism Version 7.0 according to analyze dose-response data. *∗P* < 0.05.

Lastly, in order to investigate whether IL-13 affected cell sensitivity to ADM via regulating ABCC4 expression, CCK-8 assay was conducted to detect the cytotoxic effect in ENKTL YTS cells. As shown in Figures 5(a)–5(d), cellular viability of YTS cells was repressed by ADM in a dose dependent manner. IL-13 significantly alleviated the suppressive effect of ADM on YTS cells, while knockdown of ABCC4 enhanced the suppressive effect. In addition, IL-13 treatment inversed the promotive influence of ABCC4 downregulation on cytotoxicity of ADM in YTS-sh-ABCC4 cells. Accordingly, as shown in Figure 5(e), in contrast to YTS cells, the IC50 value for ADM was found to be increased in YTS cells treated with IL-13 and reduced in YTS-sh-ABCC4 cells. Moreover, IL-13 inversed the inhibitory effect of ABCC4 downregulation on the IC50 value for ADM in YTS cells. Therefore, our results concluded that IL-13 promoted ENKTL YTS cell chemotherapy resistance to ADM by increasing ABCC4 expression.

## 4. Discussion

NK/T cell lymphomas is a rare distinct disease entity, which is a highly aggressive disease clinically [18]. Most of the cases were extranodal NK/T cell lymphoma, nasal-type (ENKTL) according to the current WHO classification scheme [19]. Patients with ENKTL are frequently perplexed by hemophagocytic syndrome, which commonly announces death [20]. Report has disclosed that ENKTL is marked by a poor outcome with only 39-64% 5-year event-free survival and 49% overall survival owing to chemoresistance [21, 22]. Because of the rarity of the disease and the difficulty in achieving adequate biopsy specimens, the multidrug resistance mechanism of ENKTL remains largely unclear.

Therefore, new alternative approaches for ENKTL are needed. Recently, the introduction of targeted therapy efficiently has improved cancer treatments. Nonetheless, even though prognosis and outcome for specific cancer patients have been certainly improved by some targeted therapies, the recurrent problem caused by therapeutic resistance has inhibited the current development of cancer therapy [23]. It is well known that P-glycoprotein (P-gp) overexpression in tumor tissues is the major mechanism of MDR, yet it is not the solely mechanism. Rising researches revealed that MDR-associated proteins (MRPs), members of ABC transporter superfamily, play important parts in cellular multidrug resistance [24, 25]. MRPs chiefly exist in the plasma membrane and seem to act as a drug pump which can reduce cellular drug levels, resulting in cellular multidrug resistance [24].

MRP4/ABCC4 is one of MRPs that are able to transport far-ranging organic compounds out of cells [26]. Abnormal expression of ABCC4 has been observed in many cancer cells and is involved in the resistance [24, 27, 28]. For example, the functions of ABCC4 have been reported in human gastric cancer, where ABCC4 expression is highly expressed in multiple types of gastric cancer cells and is even more conspicuous in the drug-resistant gastric cancer cells [29]. Moreover, ZHANG et al. also have found that suppression of ABCC4 expression can repress the proliferation of multidrug-resistant gastric cancer cells and enhance gastric cancer cell sensitivity to chemotherapeutic drugs [29]. In prostate cancer cells, OPREA-LAGER et al. have showed that the docetaxel-resistant MLL cells are found to highly express ABCC4 and that functional repression of ABCC4 in MLL cells causes a marked reduction in effective concentration of docetaxel and exhibited an improved therapeutic efficacy [30]. Recently, a study has shown that overexpression of ABCC4 is determined in human NK/T-cell lymphoma cells and regulates chemotherapy sensitivity to epirubicin (EPI) and cisplatin (DDP) in YTS cells [31]. Consistent with that, we found that ABCC4 expression level was influentially increased in ENKTL tissues and cells. Moreover, our results revealed that knockdown of ABCC4 inhibited cell proliferation and promoted apoptosis and enhanced the chemotherapy sensitivity of YTS cells to ADM. The above researches show that ABCC4 plays an important role in drug resistance in various tumors, including human ENKTL. However, the precise resistance mechanism of ABCC4 in human ENKTL remains unclear.

IL-13, a Th2 cytokine, participates in allergic reactions, fibrosis, inflammation, and even tumor development [32]. Many studies have revealed that expression level of IL-13 is elevated in various tumor tissues and peripheral blood, which is associated with poor outcome and involved in tumor progression [33]. For example, a report has shown that IL-13 may suppress cancer-directed immunosurveillance and increase tumor metastasis [33]. A study has revealed that IL-13 along with Th2 cytokines such as IL-10 and IL-4 assist in the immunological tumor escape and interfere with the induction of an antitumor response [34]. In addition, these cytokines promote the expression of c-Flip, Bcl-xL, and Mcl-1, which are related to apoptosis, and additionally facilitate cancer cells growth [34]. Moreover, IL-13 has been reported to promote proliferation of cancer cells and be related to lymph node metastasis in some malignancies [35, 36]. These studies suggest that IL-13 might be correlated with tumor growth. In our study, we found that serum level of IL-13 was obviously increased in patients with ENKTL. Interestingly, we observed that IL-13 promoted ABCC4 expression in ENKTL YTS cells. Therefore, we speculated that the increased expression level of ABCC4 induced by IL-13 may be implicated in cellular drug resistance. As was expected, IL-13 reduced the sensitivity of YTS cells to ADM and reversed the promotive effects of knockdown ABCC4 on sensitivity of YTS cells to ADM.

## 5. Conclusion

Our study showed for the first time that IL-13 inhibited chemotherapy sensitivity of ENKTL cells with promoting cell proliferation and inhibiting apoptosis of YTS cells via promoting ABCC4 expression. These findings provide a new insight into resistance mechanism of ENKTL and guide development of novel therapeutic approaches for this aggressive disease. However, this hypothesis still deserves further investigation* in vivo*.

## Data Availability

The data used to support the findings of this study are included within the article.
